# Genetics of Deafness

**DOI:** 10.1155/2012/562848

**Published:** 2012-04-18

**Authors:** Edi Lúcia Sartorato, Karen Friderici, Ignacio Del Castillo

**Affiliations:** ^1^Universidade Estadual de Campinas (UNICAMP), Centro de Biologia Molecular e Engenharia Genética (CBMEG), Campinas, 13083-970 São Paulo, Brazil; ^2^Department of Microbiology and Molecular Genetics and Pediatrics/Human Development, MSU College of Natural Science, East Lansing, MI 48824, Michigan, USA; ^3^Unidad de Genética Molecular, Hospital Ramón y Cajal, 28034 Madrid, Spain

Hearing impairment is the most common sensorineural deficit worldwide. Deafness has a major genetic component, and understanding how genetic variation impacts hearing needs to be extensively studied. The importance of this work is reflected in the review by Lufkin: “the sense of hearing is one of the most crucial senses endowed to a living organism and its loss can have many ramifications.” This special issue about the Genetics of Deafness contributes to these studies by describing new mutations in genes important in hearing, by exploring the clinical implications of treatment based on genotype as well as reviewing of the literature.

Included in this issue are reviews of genetic syndromes where deafness is a significant component. Lassaletta and colleagues set out an important review of the molecular mechanism of vestibular schwannoma. Pillion and colleagues characterize hearing loss in osteogenesis imperfecta. Tomelleri's group explores MELAS. All address molecular pathology of hearing loss and explore treatment options.

Several papers evaluate the distribution or effect of sequence variation in known hearing loss genes. Leal and colleagues describe novel mutations in two previously identified nonsyndromic hearing loss genes. Mateos et al. describe the effects of noncoding sequence variation in *GJB2*.

Vilarino and colleagues detail the distribution of mutations in pediatric patients in Portugal. Reports of this type improve our understanding of the mechanisms of hearing and the relevance of sequence variation in gene function.

Mutations in *GJB2* are the leading cause of congenital deafness in many countries. Malekpour and colleagues present a large study detailing the performance of cochlear implants in patients with and without *GJB2* mutation.

The complexity of genetic deafness and the tremendous progress associated with inherited hearing loss make it clear to us that the “silence genes” will have a lot more to tell us.

*Edi Lúcia Sartorato* *Edi Lúcia Sartorato* *Karen Friderici* *Karen Friderici* *Ignacio Del Castillo* *Ignacio Del Castillo*

